# Evaluating AI-Generated Responses for Patient Counseling and Treatment Decision-Making in Melasma: A Cross-Sectional Study

**DOI:** 10.7759/cureus.90883

**Published:** 2025-08-24

**Authors:** Shreya Deoghare, Rashmi Sarkar, Seemal R Desai

**Affiliations:** 1 Dermatology, Alexis Multi-Speciality Hospital, Nagpur, IND; 2 Dermatology, Lady Hardinge Medical College, New Delhi, IND; 3 Dermatology, The University of Texas Southwestern Medical Center, Dallas, USA

**Keywords:** artificial intelligence, counselling, decision-making, melasma, patient satisfaction

## Abstract

Background

Melasma poses challenges in treatment decisions and patient counseling due to its chronic nature and variable response to therapies. Evaluating AI-generated responses from ChatGPT (OpenAI, Inc., San Francisco, CA, USA) and ChatSonic (Writesonic, Inc., San Francisco, CA, USA) could provide insights into their potential role in supporting dermatologists with patient counseling and treatment decision-making for melasma.

Aim

This study aims to compare responses generated by ChatGPT and ChatSonic for patient counseling and treatment decisions in melasma, as evaluated by senior dermatologists for satisfaction, quality, and reliability.

Methodology

This cross-sectional observational study, conducted virtually in January 2023, involved asking five questions each about patient counseling and treatment decision-making in melasma to ChatGPT and ChatSonic. Responses were compiled in a Microsoft Excel assessment sheet (Microsoft Corporation, Redmond, WA, USA) and presented in a blinded format to two senior dermatologists (Evaluators A and B) for evaluation of quality (Global Quality Score), reliability (modified DISCERN scale), and satisfaction (Likert scale).

Results

For patient counseling in melasma, ChatGPT responses were rated higher in satisfaction by Evaluator A, with no significant differences in quality or reliability between the two AI chatbots. For treatment decisions, Evaluator B significantly favored ChatGPT over ChatSonic in both satisfaction and quality.

Conclusions

AI chatbots such as ChatGPT and ChatSonic can effectively support dermatologists in patient counseling and treatment decision-making for melasma, with ChatGPT demonstrating superior satisfaction and quality.

## Introduction

Melasma is a condition of acquired hypermelanosis that clinically presents on the face and neck as bilateral macular brown patches [1]. It has a profound negative impact on quality of life due to its prominent facial appearance, prolonged treatment duration, and high risk of recurrence. It is not unusual for an outpatient to have studied all available treatments and drug-related adverse effects before consulting a dermatologist.

Despite a global prevalence of only 1%, melasma disproportionately affects people with skin of color, such as South Asians (40%) and Latin Americans (up to 30%) [1]. Dermatologists in regions where this condition is less common may lack confidence and experience in managing melasma, potentially leading to suboptimal treatment outcomes.

The role of AI in dermatology is increasingly important, with several studies demonstrating its ability to diagnose skin lesions from clinical and dermoscopic images at levels equivalent to, or better than, dermatologists [2]. More recently, user-interactive chatbots have been developed that can generate answers to user queries based on machine learning and AI, aiming to enhance human problem-solving abilities. Applied to medical science and healthcare, these chatbots could serve as valuable tools for patient counseling and support physicians in making treatment decisions, provided they are trained with verified and accurate information.

ChatGPT is a chatbot launched by OpenAI, Inc., a San Francisco, California-based AI research and development company, in November 2022 [3,4]. It employs natural language processing and machine learning to enable conversational interactions with a virtual assistant [4,5]. ChatSonic is another AI-based user-interactive chatbot, launched by Writesonic, Inc., a San Francisco, California-based AI company, in December 2022. It has additional features such as real-time data access, image, and voice search [6,7].

In this article, we compare responses generated by two user-interactive AI chatbots, ChatGPT and ChatSonic, regarding patient counseling and treatment decisions for melasma. These responses were blinded and graded by two senior dermatologists experienced in treating pigmentary disorders in patients with skin of color, based on satisfaction, quality, and reliability.

The aim of this study was to collect responses generated by two user-interactive AI chatbots, ChatGPT and ChatSonic, by presenting each with five identical questions on melasma in the areas of patient counseling and treatment decisions, and to present these responses in a blinded format to two senior dermatologists to compare the chatbots based on satisfaction, quality, and reliability of the responses.

## Materials and methods

This cross-sectional observational study was conducted virtually in January 2023. Ethics Committee approval was not obtained, as the study did not involve any data from human participants.

Five questions were developed in two domains: (1) patient counseling, based on frequently asked questions by patients diagnosed with melasma, and (2) clinical decision-making, based on common clinical dilemmas encountered by dermatology residents when initiating treatment. The questions were designed by the primary author (SD) based on common queries received in the clinic. They were independently reviewed by two experienced dermatologists for content validity and clarity and were finalized after reaching consensus.

These questions were then posed to ChatGPT Version 3.5 [3] and ChatSonic [7] on February 20, 2023. An assessment sheet was created in Microsoft Excel (Microsoft Corporation, Redmond, WA, USA), in which the questions and the responses generated by the chatbots were compiled in a tabulated format.

Two senior board-certified dermatologists (referred to as Evaluator A and B), with substantial clinical and academic experience in pigmentary disorders in skin of color, independently evaluated the responses. They were blinded to the identity of the AI chatbot that generated each response, with all responses anonymized in the assessment sheet prepared by SD.

For grading the responses, three evaluation tools were used (Table 1). Satisfaction was graded using a Likert scale ranging from 1 to 5, where 1 indicated a highly unsatisfactory response and 5 indicated a highly satisfactory response. Quality was assessed using the Global Quality Score (GQS), ranging from 1 to 5, where 5 indicated excellent response quality [8]. Finally, reliability and accuracy were assessed using the modified DISCERN scale, a five-point scale evaluating concision, reliability, balance, referencing, and uncertainty, with higher scores indicating greater reliability [8].

**Table 1:** Evaluation of AI-generated responses using satisfaction score, modified DISCERN score, and GQS GQS, Global Quality Score

Grading for satisfaction, modified DISCERN score, and GQS
Grading “Satisfaction” of generated response: 1 – Very unsatisfactory; 2 – Unsatisfactory; 3 – Neutral; 4 – Satisfactory; 5 – Very satisfactory
Modified DISCERN score (Yes = 1, No = 0; total scores summed, highest = 5, lowest = 0): 1 – Is the aim clear, concise, and understandable?; 2 – Are sources of information reliable? (cited publications or video content from valid studies, dentists, or endodontists); 3 – Is the information presented balanced and unbiased? (includes reference to other treatment choices); 4 – Are additional sources of information provided?; 5 – Does the video address areas of uncertainty?
GQS (each question scored from 1 to 5): 1 – Poor quality, poor flow, most information missing, not useful for education; 2 – Generally poor quality and flow, limited use to the user because only some information is present and many important topics are missing; 3 – Moderate quality, suboptimal flow, somewhat useful to the user as some important information is adequately discussed but other topics are poorly covered; 4 – Good quality, generally good flow, useful to the user because most relevant information is covered but some topics are missing; 5 – Excellent quality and flow, highly useful to the user

All statistical analyses were performed using SPSS Statistics software (version 16.0; SPSS, Inc., Chicago, IL, USA). Descriptive statistics were used to summarize the satisfaction scores, modified DISCERN scores for reliability, and GQS. To compare the proportion of favorable ratings (scores of 4 or 5) between ChatGPT and ChatSonic, a two-proportion z-test was employed. This test assesses whether there is a statistically significant difference between two independent proportions. For each comparison, the z-value represents the magnitude of the difference in proportions relative to the variability expected by chance, while the p-value indicates the probability that such a difference could occur randomly. A p-value of <0.05 was considered statistically significant.

## Results

The AI software generated realistic and intelligent-sounding responses to user queries. In addition, ChatSonic provided references for its responses.

Responses generated by AI tools for patient counselling for melasma

Table 2 presents the grading assigned by Evaluator A and Evaluator B for responses generated by ChatGPT and ChatSonic regarding patient counselling for melasma. Only favorable ratings (scores of 4 and 5 on a 1-5 scale) are included in this table for all three parameters.

**Table 2:** Comparison of favorable grading by Evaluator A and Evaluator B for responses generated by ChatGPT and ChatSonic for patient counselling on melasma Asterisks (^*^) indicate statistical significance (p < 0.05). GQS, Global Quality Score

Parameter	ChatGPT	ChatSonic	p-Value (z test)	Z value
Satisfaction level (4 = Satisfactory, 5 = Highly satisfactory)				
Evaluator A	5 (100%)	2 (40%)	0.03^*^	2.07
Evaluator B	4 (80%)	2 (40%)	0.057	1.89
Reliability score (4 = High, 5 = Very high)				
Evaluator A	0 (0%)	1 (20%)	0.29	-1.05
Evaluator B	0 (0%)	2 (40%)	0.11	-1.58
GQS (4 = Good, 5 = Excellent quality)				
Evaluator A	3 (60%)	1 (20%)	0.19	1.29
Evaluator B	3 (60%)	2 (40%)	0.52	0.63

Satisfaction Level

Evaluator A rated a higher percentage of responses from ChatGPT as “Satisfactory” or “Highly satisfactory” (100%) compared to ChatSonic (40%), a difference that was statistically significant (p = 0.03). Evaluator B also rated a higher percentage of ChatGPT responses as “Satisfactory” or “Highly satisfactory” (80%) compared to ChatSonic (40%), but this difference was not statistically significant (p = 0.057).

Reliability Score

Both evaluators rated a lower percentage of ChatGPT responses as reliable (0%) compared to ChatSonic (20% for Evaluator A and 40% for Evaluator B), with reliability defined as “High” to “Very high.” These differences were not statistically significant (p = 0.29 for Evaluator A, p = 0.11 for Evaluator B).

Quality (GQS Score)

Evaluator A rated a higher percentage of ChatGPT responses as “Good” or “Excellent” quality (60%) compared to ChatSonic (20%), though this difference was not statistically significant (p = 0.19). Evaluator B rated an equal percentage of ChatGPT and ChatSonic responses as “Good” or “Excellent” quality (60% each), with no statistically significant difference (p = 0.52).

Overall, the findings suggest that Evaluator A tended to rate ChatGPT responses more favorably than ChatSonic, particularly in terms of satisfaction. Evaluator B showed less consistent preferences between the two chat systems, with differences in ratings but fewer statistically significant findings.

Responses generated by AI tools for treatment decision-making in melasma

Table 3 presents the favorable grading assigned by Evaluator A and Evaluator B for responses generated by ChatGPT and ChatSonic regarding treatment decision-making for melasma. Only favorable ratings (scores of 4 and 5 on a 1-5 scale) are included in this table for all three parameters.

**Table 3:** Comparison of favorable grading by Evaluator A and Evaluator B for responses generated by ChatGPT and ChatSonic for making treatment decisions on melasma Asterisks (^*^) indicate statistical significance (p < 0.05). GQS, Global Quality Score

Parameter	ChatGPT	ChatSonic	p-Value (z test)	Z value
Satisfaction level (Satisfactory and Highly satisfactory)				
Evaluator A	4 (80%)	5 (100%)	0.29	-1.05
Evaluator B	5 (100%)	1 (20%)	0.009^*^	2.52
Reliability score (4 = High, 5 = Very High)				
Evaluator A	0 (0%)	1 (20%)	0.29	-1.05
Evaluator B	1 (20%)	2 (40%)	0.49	-0.69
GQS (4 = Good, 5 = Excellent Quality)				
Evaluator A	4 (80%)	3 (60%)	0.49	0.69
Evaluator B	3 (60%)	0 (0%)	0.03^*^	2.07

Satisfaction Level

Evaluator A rated a lower percentage of ChatGPT responses as “Satisfactory” or “Highly satisfactory” (80%) compared to ChatSonic (100%); however, this difference was not statistically significant (p = 0.29). Evaluator B rated a higher percentage of ChatGPT responses as “Satisfactory” or “Highly satisfactory” (100%) compared to ChatSonic (20%), a difference that was statistically significant (p = 0.009).

Reliability Score

Both evaluators rated a lower percentage of ChatGPT responses as having “Very high” reliability (0%) compared to ChatSonic (20% for Evaluator A and 40% for Evaluator B). These differences were not statistically significant (p = 0.29 for Evaluator A and p = 0.49 for Evaluator B).

Quality (GQS Score)

Evaluator A rated a higher percentage of ChatGPT responses as “Good” or “Excellent” quality (80%) compared to ChatSonic (60%), though this difference was not statistically significant (p = 0.49). Evaluator B rated a higher percentage of ChatGPT responses as “Good” or “Excellent” quality (60%) compared to ChatSonic (0%), a difference that was statistically significant (p = 0.03).

Overall, the findings suggest that Evaluator A and Evaluator B displayed different patterns in rating responses from ChatGPT and ChatSonic for treatment decision-making in melasma. Evaluator A tended to rate responses from both chatbots similarly across most criteria, while Evaluator B showed more variation, with some statistically significant differences favoring ChatGPT.

## Discussion

This cross-sectional study aimed to compare responses generated by ChatGPT and ChatSonic regarding patient counselling and treatment decisions for melasma. For patient counselling, Evaluator A rated responses generated by ChatGPT more favorably than those from ChatSonic, particularly in terms of satisfaction levels. Evaluator B, however, showed less consistent preferences. Regarding questions related to treatment decisions for melasma, Evaluator B favored responses from ChatGPT, whereas Evaluator A rated responses from both chatbots similarly.

Several studies have assessed responses generated by ChatGPT and other AI tools for common dermatological conditions such as sunscreen use [9], melanoma [9,10], acne vulgaris [4,11], atopic dermatitis [11], rosacea [12], and even physician queries [13]. Research on pigmentary disorders remains limited despite their significant prevalence and impact on quality of life. This study was undertaken to evaluate AI chatbot responses for patient counselling and treatment decisions in melasma.

Mondal et al. evaluated responses generated by ChatGPT for fourteen dermatological conditions [14]. Similar to our findings, they reported that ChatGPT can generate easily understandable paragraphs suitable for patient education [14]. Goodman et al. reported high accuracy of ChatGPT-generated responses to common patient queries using a Likert scale [14]. In our study, quality was assessed using the GQS, with more responses from ChatGPT rated as good or excellent compared to ChatSonic.

Yan et al. concluded that ChatGPT 3.5 demonstrates strong reliability and clinical utility in addressing common patient inquiries regarding rosacea, making it a valuable tool for enhancing patient education on this condition [12]. Our study, in contrast, found that both evaluators rated a lower percentage of ChatGPT responses as reliable compared to ChatSonic for both patient counselling and treatment decisions. This difference may be due to our use of a modified DISCERN score to evaluate reliability (Table 1), whereas Yan et al. used a Likert scale from 1 (completely unreliable) to 5 (completely reliable).

Although ChatGPT can be beneficial for patient education and clinical decision-making, it cannot provide comprehensive diagnoses and lacks essential human qualities required in medical practice [15].

This study had several limitations. First, only five questions were used for each domain (patient counselling and treatment decisions). While these questions reflected common queries about melasma, a larger set would provide a more comprehensive representation. Second, responses were evaluated by only two evaluators; including more evaluators would improve the robustness and reliability of the findings. Additionally, responses generated by ChatGPT and ChatSonic are not entirely consistent; they may vary when the same questions are asked repeatedly or with minor modifications. This variability was not accounted for in our study, which may affect the generalizability and applicability of the results. Future studies should include a broader range of questions and more evaluators to ensure a more accurate and reliable assessment.

## Conclusions

This study demonstrates that AI chatbots such as ChatGPT and ChatSonic can provide valuable responses for patient counselling and treatment decisions in melasma. ChatGPT was rated more favorably in satisfaction and quality by one evaluator. These findings underscore the potential of AI tools to support dermatologists, particularly in regions with limited experience in treating melasma. However, the study’s limitations, including a small set of questions and a limited number of evaluators, highlight the need for future research with a broader scope and more evaluators to better assess the consistency and applicability of AI-generated responses in dermatological care.
